# Sleep and Thermoregulation in Birds: Cold Exposure Reduces Brain Temperature but Has Little Influence on Sleep Time and Sleep Architecture in Jackdaws (*Coloeus monedula*)

**DOI:** 10.3390/biology13040229

**Published:** 2024-03-29

**Authors:** Sjoerd J. van Hasselt, Massimiliano Coscia, Giancarlo Allocca, Alexei L. Vyssotski, Peter Meerlo

**Affiliations:** 1 Groningen Institute for Evolutionary Life Sciences, University of Groningen, 9747 AG Groningen, The Netherlands; 2 School of Biomedical Sciences, University of Melbourne, Parkville, VIC 3010, Australia; 3 Somnivore Pty. Ltd., Bachhus Marsh, VIC 3340, Australia; 4 Institute of Neuroinformatics, University of Zurich and Swiss Federal Institute of Technology (ETH), 8057 Zurich, Switzerland

**Keywords:** birds, corvids, EEG, sleep, REM sleep, brain temperature, ambient temperature, cold exposure

## Abstract

**Simple Summary:**

Birds have an electrophysiological sleep state that resembles rapid-eye-movement (REM) sleep in mammals. Whether the regulation and function of this REM sleep state in birds is similar to that in mammals is unclear. In the current experiment, we studied sleep regulation in jackdaws (*Coloeus monedula*) by exposing the birds to low ambient temperature, a procedure that selectively suppresses REM sleep in mammals. Jackdaws were equipped with electrodes to record brain activity and neck muscle activity and a sensor to record brain temperature. Exposure to a low ambient temperature of 4 °C during the nighttime sleep phase caused a significant drop in brain temperature of 1.4 °C, compared to the baseline night at 21 °C. However, cold exposure did not affect the amount of NREM sleep and REM sleep. This indicates that REM sleep in jackdaws is protected against the influence of low temperature. Hence, the well-established relationship between thermoregulation and REM sleep regulation that exists in mammals may not be present in birds.

**Abstract:**

Birds have an electrophysiological sleep state that resembles mammalian rapid-eye-movement (REM) sleep. However, whether its regulation and function are similar is unclear. In the current experiment, we studied REM sleep regulation in jackdaws (*Coloeus monedula*) by exposing the birds to low ambient temperature, a procedure that selectively suppresses REM sleep in mammals. Eight jackdaws were equipped with electrodes to record brain activity and neck muscle activity and a thermistor to record cortical brain temperature. Recordings covered a three-day period starting with a 24 h baseline day at an ambient temperature of 21 °C, followed by a 12 h cold night at 4 °C, after which the ambient temperature was restored to 21 °C for the remaining recovery period. Cold exposure at night caused a significant drop in brain temperature of 1.4 °C compared to the baseline night. However, throughout the cold night, jackdaws expressed NREM sleep and REM sleep levels that were not significantly different from the baseline. Also, EEG spectral power during NREM sleep was unaffected by cold exposure. Thus, while cold exposure had a clear effect on brain temperature in jackdaws, it did not have the same REM sleep suppressing effect reported for mammals. These findings suggest that the REM-sleep-like state in birds, unlike REM sleep in mammals, is protected against the influence of low temperature.

## 1. Introduction

While sleep is thought to be a widespread phenomenon that occurs in nearly all animals, much of our knowledge on the regulation and function of sleep is derived from studies in nocturnal rodents [1]. Studying organisms outside of the mammalian class can provide important insights into the evolution of sleep that can either confirm or expand on current theories regarding sleep function [1,2,3,4]. Birds are an interesting group of animals for studying sleep because they display two electrophysiologically distinct sleep states that are similar to mammalian rapid-eye-movement (REM) sleep and non-REM (NREM) sleep [5,6,7]. The pattern of brain activity (electroencephalogram, EEG) during the NREM sleep state is characterized by high-amplitude slow waves, whereas the EEG during the REM sleep state is characterized by low-amplitude fast waves. Also, in agreement with studies in mammals, there is some evidence that NREM sleep in birds is homeostatically regulated, as reflected in compensatory rebounds after sleep deprivation [8,9,10] even though this response may be modulated by environmental conditions and season [9].

On the other hand, the expression of REM sleep is more variable among birds than it is among mammals, ranging from less than 1 to 25% of total sleep time [11]. Furthermore, the episode length of REM sleep is generally shorter in birds than in mammals [11,12]. In fact, findings in various bird species challenge the current definitions of REM sleep, including the finding that some birds lack the full muscle atonia that is typically seen during REM sleep in mammals [11,13,14,15]. Such findings raise the question whether REM sleep in mammals and birds is mechanistically and functionally the same state. Examining the effects of selective manipulation of REM sleep could provide insights into the underlying mechanisms of this sleep state and whether or not these are similar in birds and mammals.

The expression of REM sleep in mammals is known to be highly sensitive to environmental temperature [16,17]. In particular, studies in several different species of mammals have shown that exposure to low ambient temperature is associated with a strong and selective reduction in REM sleep, including in rats (*Rattus norvegicus*) [18,19,20], cats (*Felis catus*) [21], and tupaias (*Tupaia belangeri*) [22]. The cold-induced suppression of REM sleep is followed by a REM sleep rebound during subsequent recovery days at thermoneutral temperatures in some species [18,19,20] but not in all [22].

The cold-induced suppression of REM sleep in mammals may protect their brains against uncontrolled cooling that would result from the remarkable loss of thermoregulation that occurs during this state [16,17]. Several studies have shown that thermoregulatory responses that occur during wakefulness and NREM sleep, such as shivering and panting, are attenuated or absent during REM sleep [16,23].

Given the clear and selective REM sleep suppressing effects of low ambient temperature in mammals, cold exposure can be used as an experimental approach to assess if REM sleep mechanisms in birds are similar to those in mammals. However, knowledge on the influence of ambient temperature on REM sleep regulation in birds is limited. Studies in rooks (*Corvus frugilegus*) and magpies (*Pica pica*) have reported lower amounts of REM sleep under conditions of shorter photoperiod and lower environmental temperatures, but it is unclear how much of the decrease in REM sleep is due to photoperiod and temperature [24]. Studies in emperor penguins (*Aptenodytes forsteri*) showed no difference between cold (−17 °C) and thermoneutrality (−8 °C) in total sleep time and REM sleep fraction [25].

In order to increase our knowledge on REM sleep regulation in birds, we performed an experimental study on the effects of cold exposure in the European jackdaw (*Coloeus monedula*). The jackdaw is a suitable model organism since it is a bird species with high amounts of baseline REM sleep, similar to the amounts reported for mammals [26]. Moreover, it is a species in which the regulation and expression of sleep was shown to be highly sensitive to environmental influences such as light [26].

## 2. Materials and Methods

### 2.1. Animals and Housing

Eight European jackdaws were used in this study (4 males, 4 females). The birds were retrieved as 30-day-old nestlings from nest boxes in a wild jackdaw colony. The young birds were transported to the animal facility of our research institute and were then group-housed, separated by sex in two seminatural outdoor enclosures (length = 5 m, width = 4 m). The birds were hand-fed 7 times a day between sunrise and sunset for a period of 45 days (Versele-Laga, NutriBird A21, Deinze, Belgium). At the age of 2 months, they slowly acclimated to regular tap water and commercial food pallets (food item number 6659; Kasper Faunafood, Woerden, The Netherlands). At the age of 10 months, they underwent surgery for implantation of a thermistor to record cortical brain temperature and electrodes for electroencephalogram (EEG) and electromyogram (EMG). Two weeks prior to the start of the experiment, the animals were moved indoors into individual wooden cages (length = 79 cm, width = 60 cm, height = 60 cm) with wood shavings as bedding material and a wooden perch to sit on. Each cage contained two light bulbs that provided a 12:12 light–dark cycle (lights on at 8 a.m.). All the cages were placed in a climate-controlled room that had a temperature kept at 21 °C, except for the experimental night when the temperature in the room was decreased to 4 °C. Food and water were present ad libitum throughout the experiment. All procedures were approved by the national Central Authority for Scientific Procedures of Animals (CCD) and the local Institutional Animal Welfare Body (IvD) at University of Groningen, The Netherlands (project number AVD10500202115448).

### 2.2. Surgery

Prior to surgery, birds received an intramuscular injection of meloxicam (0.022 mL, 0.5 mg/kg) that served as an analgesic. Subsequently, anesthesia was induced by isoflurane at 5% (O_2_ 0.3 L/min, Air 0.6 L/min) and was then maintained between 1 and 3%. Lidocaine was applied on the head as a local anesthetic. After carefully exposing the cranium, 4 holes with a diameter of 0.5 mm were drilled, and rounded gold-plated electrode pins (BKL Electronics 10120538, Lüdenscheid, Germany) were inserted at the level of the dura mater. Two frontal holes were drilled 2 mm lateral of the midline over the rostral part of the hyperpallium and served as EEG electrodes. The two remaining holes were drilled 2 mm lateral of the midline over the caudal part of the hyperpallium near the cerebellum and served as a reference and ground electrode. Additionally, 2 holes with a diameter of 1 mm were drilled to the level of the dura mater at 4 mm caudal and 4 mm lateral of the frontal electrodes for placement of a thermistor (TE Electronics Ltd., GA10K3MCD1, Schaffhausen, Switzerland) and an anchor screw (1.2 mm in diameter) on the left and right hemisphere, respectively. Lastly, a flexible wire was placed on the neck muscle to measure electromyogram (EMG). All EEG and EMG electrodes and the thermistor electrodes were soldered to a 7-channel connector (BKL Electronic 10120302, Lüdenscheid, Germany). All electrodes and the connector were then secured using dental acrylic (Paladur, Heraeus Kulzer, Hanau, Germany). During the recovery period and in between recordings, the connector was covered with a light-weight protective plug (BKL Electronic 10120602, Lüdenscheid, Germany).

### 2.3. Data Collection

Miniature dataloggers (Neurologger 2A, Evolocus, Tarrytown, NY, USA) were used to record and store EEG, EMG, and thermistor data as well as head movements by an onboard three-axis accelerometer (LIS302DLH; STMicro-electronics, Geneva, Switzerland). The dataloggers were powered by two ZA13 1.45 V zinc–air batteries (Ansmann ZA13, Assamstadt, Germany) and could record up to 5 days with a sampling rate of 100 Hz. Data collection lasted for a total of 72 h and consisted of a 24 h undisturbed baseline recording, a 12 h nighttime cold exposure during which the ambient temperature was lowered from 21 °C to 4 °C, and a subsequent 36 h of recovery with the ambient temperature restored to 21 °C.

### 2.4. Data Analysis

**Figure 1:**
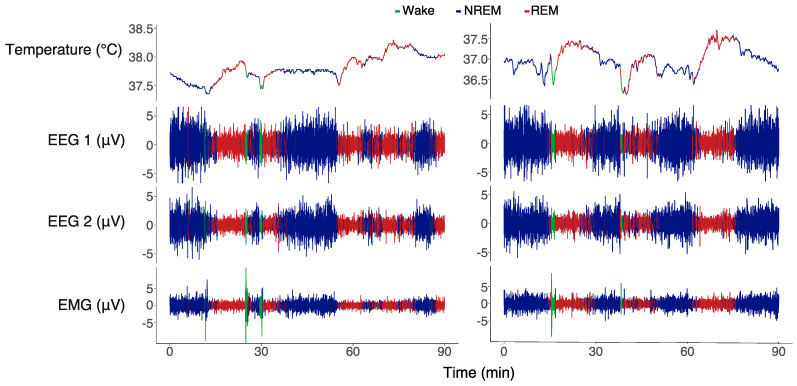
Two 90 min examples of a baseline recording (**left**) and a cold exposure recording (**right**) during the same time of the night. The graphs display brain temperature, EEG 1 (left hyperpallium), EEG 2 (right hyperpallium), and the EMG trace. The different colors of the traces indicate the vigilant states: blue denotes NREM sleep, red denotes REM sleep, and green denotes wakefulness. NREM sleep was characterized by slow-frequency and high-amplitude signals with moderate EMG activity. REM sleep was characterized by high-frequency and low-amplitude signals with reduced EMG activity. Wakefulness was characterized by high-frequency and low-amplitude EEG signals and activity in the EMG.

All recorded data were automatically scored for wakefulness, NREM sleep, and REM sleep on a 4 s basis using a machine-learning algorithm (Somnivore Pty. Ltd., Parkville, VIC, Australia) [27]. The program uses all available electrophysiological channels (EEG + EMG + accelerometer) to determine the vigilance state. Wakefulness was scored when there were high-frequency and low-amplitude waves in combination with high EMG and accelerometer activity. NREM sleep was scored when the EEG showed slow frequencies and high-amplitude waves in combination with low EMG activity and no accelerometer movements. REM sleep was scored when the EEG displayed high-frequency and low-amplitude waves in combination with reduced EMG activity and low to no accelerometer movements (Figure 1). The program was trained for every recording by an experienced scorer that manually scored ~100 epochs per vigilance state. The automated scoring program has been validated for several species of mammals and birds, including pigeons, which resulted in a scoring accuracy of 0.96 ± 0.01 for wakefulness, 0.97 ± 0.01 for NREM sleep, and 0.86 ± 0.02 for REM sleep as compared to a manually scored recording [27]. We conducted a further validation of the scoring for our recordings in jackdaws on a subset of 4 baseline recordings that yielded an accuracy of 0.98 ± 0.02 for Wake, 0.95 ± 0.02 for NREM sleep, and 0.89 ± 0.03 for REM sleep.

After sleep scoring, we conducted another round of artifact scoring to obtain clean EEG data. Artifacts were scored when the EEG showed an amplitude twice that of an undisturbed signal in combination with high EMG and accelerometer activity. A Fast Fourier Transformation (FFT) was performed on clean NREM sleep EEG data. The FFT yielded 256 frequency bins with a bandwidth of ~0.2 Hz. For each frequency bin, all FFT power values were normalized to the average nighttime baseline power. The normalized power levels in the frequency range of 1.5–25 Hz were averaged. This broad frequency range has been suggested to reflect NREM sleep homeostasis in songbirds [8,10]. These power levels were further averaged per hour, and the cumulative NREM sleep energy was calculated as the product of NREM sleep power and NREM sleep time.

Furthermore, every individual brain temperature recording was averaged per minute and expressed as deviation from the individual’s average 24 h baseline to correct for interindividual differences in the temperature signal. Additionally, to determine the relative changes in nighttime brain temperature across episodes of different vigilance states, the 1 s temperature values in each episode were expressed relative to the temperature at the start of the episode. To deal with circadian modulation of brain temperature (Tbr) in this analysis, we applied a first-order bandpass Butterworth filter that removed frequencies above 0.000012 Hz (i.e., waves with a period larger than 23.15 h) using the R package eegkit [28]. The filtering successfully eliminated circadian temperature fluctuations while retaining the faster stage-dependent fluctuations (Figure S1 and Figure 1).

### 2.5. Statistics

Data were processed and analyzed in R [29]. To test the effect of cold exposure on NREM and REM sleep time, we used a linear mixed effect model using the lme4 package. We performed several lmer models where we tested the relationship between the outcome variables brain temperature, NREM/REM sleep time, NREM sleep EEG intensity, and cumulative EEG energy with the predictors day (baseline, cold exposure + 1st recovery, 2nd recovery) and time of the day (in hours) including a random component of bird identity [30]. Due to non-linearity, we used general additive modeling (gam) to test the relationship between the outcome variable relative brain temperature with the predictors episode length (in seconds), vigilant state (NREM, REM, and wakefulness), and day (baseline, cold exposure, 2nd recovery) using the mgcv package [31]. The significance of predictors was further tested by the AIC index and the type 2 ANOVA test to reach the minimum adequate model. Statistical differences between groups from the lmer and gam minimum adequate models were calculated using the Tukey HSD post hoc test from the emmeans package [32].

## 3. Results

Figure 1 shows two 90 min examples of recordings of brain temperature (Tbr), EEG, and EMG during a baseline night (left panel) and cold exposure night (right panel). The NREM sleep episodes (blue) in this example had an EEG amplitude about twice as high as that during REM sleep and wakefulness. REM sleep episodes (red) were associated with reduced muscle activity, as reflected in the EMG channel. The brief nighttime Wake episodes (green) were associated with instant activation of the neck muscle EMG.

**Figure 2:**
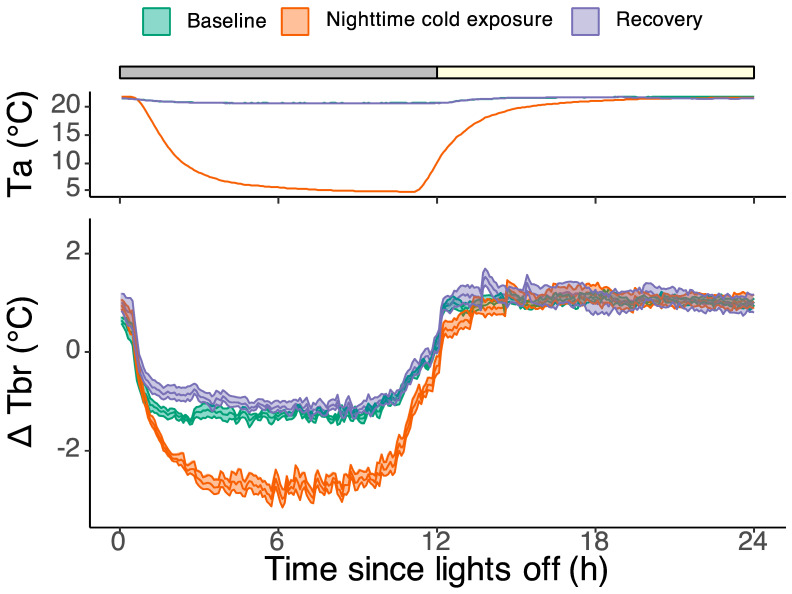
The top panel denotes the ambient temperature inside the wooden enclosures during the baseline day (green), the experimental day with nighttime cold exposure (orange), and the recovery day (purple). The grey and yellow bar on top denotes the dark and light phases, respectively. During the baseline and recovery day, the ambient temperature was 21.3 ± 0.03 °C. At the start of the cold exposure night, the ambient temperature gradually decreased from 21 °C to a minimum of 4.8 °C. Two hours prior to lights on, the ambient temperature started rising again and reached baseline levels in the middle of the light phase. The bottom panel shows the brain temperature during the same three days, expressed as deviation from the average 24 h baseline temperature. The shaded area around the lines indicates the SEM. During the 24 h baseline day, the brain temperature was 2.1 ± 0.12 °C lower during the night compared to the day (*p* < 0.001, lmer model). Cold exposure at night induced a further drop of 1.1 ± 0.2 °C (*p* < 0.001, lmer model). The maximum drop during the night was 1.4 ± 0.2 °C below baseline. During the subsequent light phase, the brain temperature was not significantly different from baseline levels (*p* = 0.8, lmer model).

Under baseline conditions with a constant ambient temperature of 21 °C, the Tbr was 2.1 ± 0.12 °C lower during the dark phase compared to the light phase (*p* < 0.001, lmer model; Figure 2). When the ambient temperature in the experimental room was lowered to 4 °C during the next night, on average, the Tbr dropped by 1.1 ± 0.2 °C below baseline values (*p* < 0.001, lmer model). The maximum drop during the night was on average 1.4 ± 0.2 °C. During the subsequent light phase, when the room temperature was restored to 21 °C, the Tbr returned to baseline levels (*p* = 0.8; lmer model). During the next recovery night, the Tbr was similar to baseline values (*p* = 0.35; lmer model).

**Figure 3:**
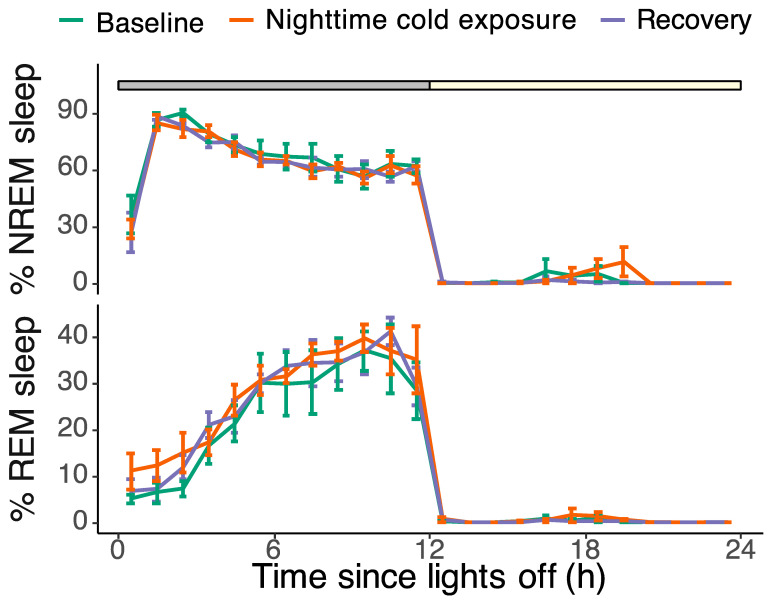
Hourly percentages of NREM sleep (top panel) and REM sleep (bottom panel) during the baseline day (green), the experimental day with nighttime cold exposure (orange), and the recovery day (purple). The grey and yellow bar on top denotes the dark and light phases, respectively. Most sleep occurred during the 12 h dark period, with little to no sleep during the light phase. The amount of NREM sleep gradually decreased over the course of the night, whereas the amount of REM sleep increased. Cold exposure had no effect on the hourly expressions of both NREM and REM sleep (NREM: F_2,337_ = 0.30, *p* = 0.74; REM: F_2,337_ = 0.57, *p* = 0.6; lmer model). Data are plotted as mean ± SEM.

The baseline EEG recordings showed a pronounced daily rhythm in sleep and wakefulness, with most of the sleep occurring during the night (90.5 ± 1.3% of the 12 h dark phase vs. 1.6 ± 1.4% during the 12 h light phase). REM sleep made up 26 ± 0.4% of the total sleep time during the dark phase, 24 ± 1.6% during the light phase, and 26 ± 0.4% during the total 24 h baseline day. Although cold exposure had a highly significant effect on brain temperature, it did not affect the overall amount of sleep during the cold night. The amount of NREM and REM sleep during the cold night was not different from the baseline (NREM: F_2,337_ = 0.30, *p* = 0.74; REM: F_2,337_ = 0.57, *p* = 0.6; lmer model; Figure 3).

**Figure 4:**
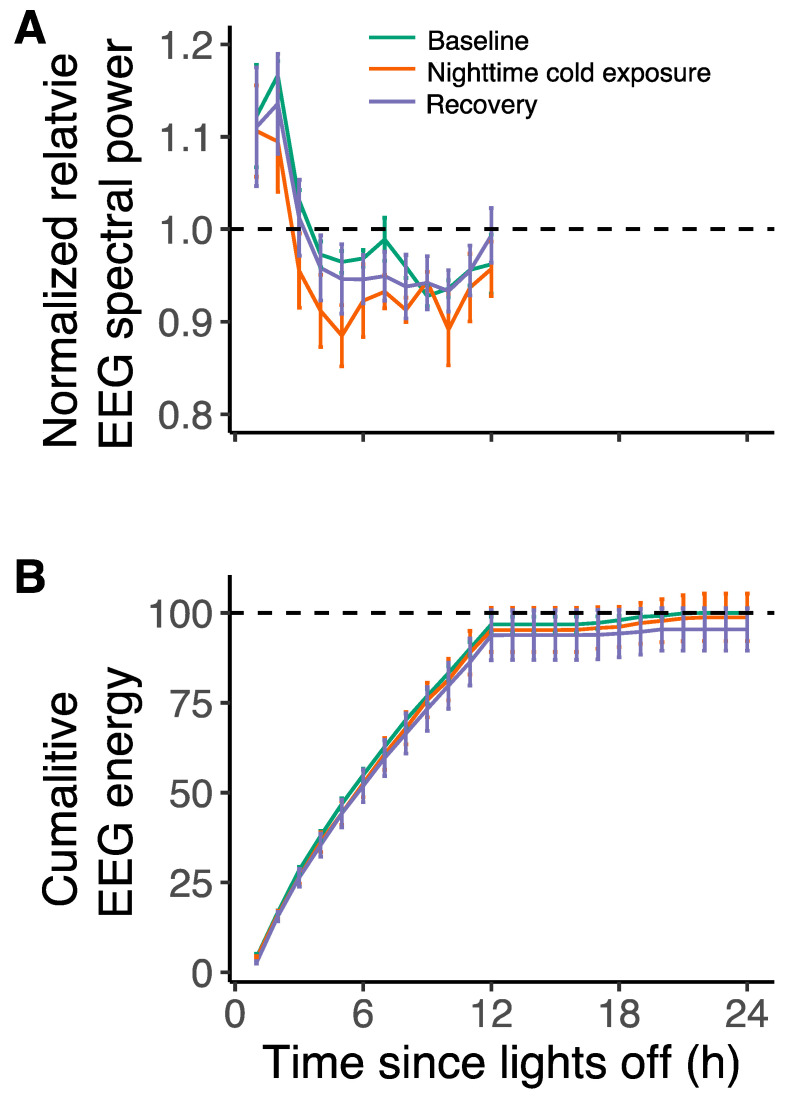
(**A**) Normalized EEG spectral power relative to average baseline for NREM sleep during the baseline day (green), the experimental day with nighttime cold exposure (orange), and the recovery day (purple). NREM sleep power was highest in the first two hours of the night, after which it steeply declined. There were no differences in EEG power between the three days (F_2,248_ = 1.41, *p* = 0.25, lmer model). (**B**) Cumulative EEG energy of NREM sleep during the three days. There were no differences in EEG energy for NREM sleep between the three days (F_2,423_ = 1.62, *p* = 0.20, lmer model). Data are plotted as mean ± SEM.

Besides sleep time, we analyzed the data for the effects of cold exposure on NREM sleep EEG spectral power. NREM sleep EEG power in the 1.5–25 Hz frequency range was highest during the first two hours of the night and rapidly declined over the course of the night. Cold exposure did not change the nighttime NREM sleep EEG power (F_2,248_ = 1.41, *p* = 0.25; lmer model, Figure 4A). Furthermore, the NREM sleep energy, i.e., the product of NREM sleep time and NREM sleep EEG power, was not affected by cold exposure either (F_2,423_ = 1.62, *p* = 0.20; lmer model; Figure 4B).

**Figure 5:**
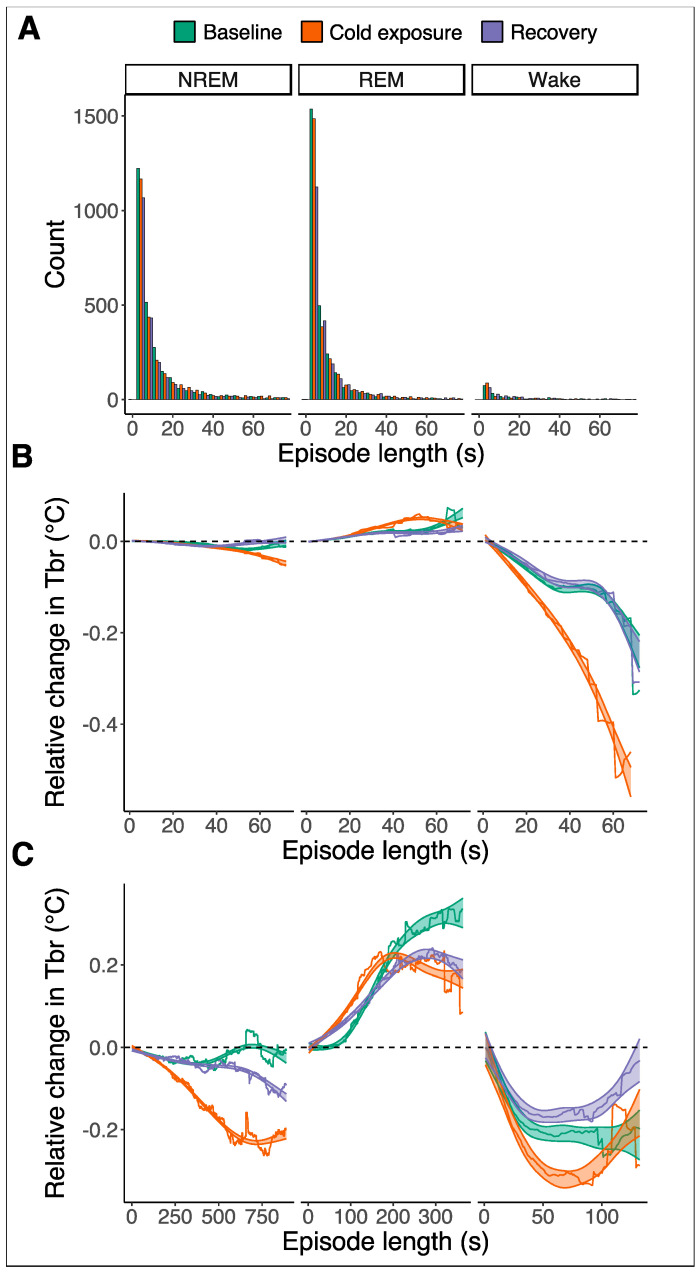
(**A**) Histograms representing the episode length of NREM sleep (left), REM sleep (middle), and wakefulness (right) during the baseline night (green), the cold exposure night (orange), and the next recovery night (purple). The episode lengths of the three vigilant states do not differ between the three recording nights. The average episode lengths were 31.8 ± 4.9 s for NREM sleep, 11.1 ± 1.0 s for REM sleep, and 50.3 ± 13.6 s for Wake. During the cold exposure night, the average episode length was 34.2 ± 7.7 s for NREM sleep, 15.1 ± 2.4 s for REM sleep, and 52.0 ± 10.6 s for Wake. During the second recovery night, the average episode length was 28.9 ± 3.4 s for NREM sleep, 16.1 ± 2.3 s for REM sleep, and 50.1 ± 11.0 s for Wake. (**B**) Relative changes in brain temperature (Tbr) during the three recording nights where the episodes were shorter than 75 s. There are little but significant non-linear changes in Tbr during episodes of NREM sleep and REM sleep for the three nights (F_2_ = 23, *p* < 0.001 and F_2_ = 41, *p* < 0.001, respectively; gam model). For NREM sleep episodes up to 75 s in length, Tbr showed small but significant non-linear fluctuations that were also statistically different between the nights (post hoc test after gam model, *p* < 0.001 for all nights). During the cold night, there was a significantly steeper increase in Tbr during REM episodes compared to the baseline and recovery night (*p* < 0.001, post hoc test after gam model), but due to an early plateauing, it did not result in a higher relative increase. During episodes of wakefulness, the Tbr decreases. This decrease is further enhanced during the cold exposure (F_2_ = 351, *p* < 0.001; post hoc test after gam model). For the REM and Wake episodes, there were no significant differences between the baseline and next recovery night (*p* = 0.6 and *p* = 0.18, respectively; post hoc test after gam model). (**C**) Relative changes in Tbr during the three recording nights for episodes of NREM, REM, and wakefulness that were longer than 75 s. Changes in relative Tbr are larger during NREM and REM sleep and in opposite directions. During cold exposure, relative Tbr decreases more significantly during episodes of NREM sleep and wakefulness (F_2_ = 2165, *p* < 0.001 and F_2_ = 120, *p* < 0.001, respectively; post hoc test after gam model). Episodes of REM sleep are associated with a larger relative increase in Tbr (F_2_ = 66, *p* < 0.001; post hoc test after gam model).

Also, cold exposure had no significant effect on the average episode length of the different vigilance states (Figure 5A). During the 12 h baseline night, the average episode length was 31.8 ± 4.9 s for NREM sleep, 11.1 ± 1.0 s for REM sleep, and 50.3 ± 13.6 s for Wake. During the cold exposure night, the average episode length was 34.2 ± 7.7 s for NREM sleep, 15.1 ± 2.4 s for REM sleep, and 52.0 ± 10.6 s for Wake. During the second recovery night, the average episode length was 28.9 ± 3.4 s for NREM sleep, 16.1 ± 2.3 s for REM sleep, and 50.1 ± 11.0 s for Wake.

To assess the regulation of the Tbr across the different vigilant states during the night, we analyzed the changes in the Tbr across each episode relative to the start of the episode (Figure 5). Based on the distribution of episode lengths (Figure 5A), we performed this separately for episodes up to a maximum length of 75 s, which included the majority of episodes for each vigilance state (Figure 5B), and for the long episodes above 75 s, which happened occasionally (Figure 5C).

For NREM sleep episodes up to 75 s in length, the Tbr showed small but significant non-linear fluctuations during the three nights (F_2_ = 23, *p* < 0.001, for all three nights) that were also statistically different between the nights (Figure 5B left; post hoc test after gam model, *p* < 0.001 for all nights). During the baseline and recovery night, NREM sleep-associated temperature changes were less than −0.03 °C. During cold exposure, the Tbr on average dropped by −0.05 °C relative to the start of the NREM sleep episode. For the occasional longer NREM sleep episodes, the Tbr gradually decreased in the cold night further to −0.23 °C below the starting temperature (Figure 5C left; gam model, F_2_ = 2165, *p* < 0.001).

The bulk of REM sleep episodes up to 75 s in length were associated with a significant but small non-linear increase in Tbr of up to 0.06 °C above the starting temperature during the baseline night and the cold night and an increase up to 0.03 °C during the recovery night (Figure 5B middle; gam model, F_2_ = 41, *p* < 0.001 for all nights). During the cold night, there was a significant steeper increase in the Tbr during REM episodes compared to the baseline and recovery night (*p* < 0.001, post hoc test after gam model), but due to early plateauing, it did not result in a higher maximum. There were no significant differences between the baseline and recovery night in relative Tbr for REM sleep episodes (Figure 5B middle; post hoc test after gam model, *p* = 0.6). During the occasional longer REM sleep episodes, the Tbr increased more substantially, up to a maximum of 0.33 °C during the baseline night and up to 0.22 °C during cold exposure and recovery nights (Figure 5C middle; gam model, F_2_ = 66, *p* < 0.001 for all nights). These increases in the Tbr were steeper during the cold night compared to the baseline and recovery night but did not reach the same maximum as during the baseline night (post hoc test after gam model, cold versus baseline: *p* = 0.02; cold versus recovery: *p* < 0.001).

The Wake episodes up to 75 s in length were associated with a steady drop in the Tbr down to 0.23 °C below the starting temperature during the baseline and recovery night (gam model, F_2_ = 351, *p* < 0.001 for both nights); yet, there were no significant differences between the nights (Figure 5B right; post hoc test after gam mode, *p* = 0.18). A much stronger drop in the Tbr of around 0.52 °C below the starting temperature was observed during waking episodes in the cold night (Figure 5B right; gam model, F_2_ = 351, *p* < 0.001), which was significantly below the baseline and recovery night (post hoc test after gam model, *p* < 0.001 for both comparisons). The longer Wake episodes with a duration above 75 s showed the same initial drop in temperature. This drop was 0.1 °C stronger during the cold night than during the baseline night, but in the later part of these long Wake episodes, the Tbr gradually plateaued and then increased (Figure 5C right; gam model, F_2_ = 120, *p* < 0.001 for both baseline and cold nights). 

## 4. Discussion

In this study, we assessed the dynamic relationship between the ambient temperature, brain temperature, and sleep in a corvid species. We show that lowering the ambient temperature to well below the thermal neutral zone caused a significant drop in the brain temperature but did not change the sleep time, sleep architecture, and NREM sleep EEG spectral power. Importantly, while exposure to cold results in a strong and largely selective suppression of REM sleep in mammals, this was not the case in our birds.

Under baseline conditions, the jackdaws had clear and parallel daily rhythms in brain temperature and sleep–wakefulness, with a high brain temperature and most wakefulness during the light phase and low temperatures and most sleep during the night phase. The difference in daytime and nighttime brain temperature was a little over 2 °C, which is in agreement with reports on the rook, another corvid species [33].

The birds spent 90.5% of the dark phase asleep, of which 74% was NREM sleep and 26% was REM sleep. During the light phase, the birds were asleep for only 1.6% of the time, with largely similar fractions of NREM and REM sleep as during the night. This pattern of predominantly nighttime sleep, as well as the proportions of NREM and REM sleep, are in agreement with our earlier study in jackdaws [26]. However, the later study showed that the overall amount of sleep strongly varies with season, i.e., the length of the night [26].

In the current study, we assessed the effects of the ambient temperature on sleep and temperature regulation. We exposed jackdaws to a low ambient temperature of 4 °C. According to published data on the effect of ambient temperature and resting metabolic rate in many different songbird species, including the jackdaw, an ambient temperature of 4 °C is on average between 4.5 °C and 9.7 °C below the thermoneutral zone in winter and summer, respectively [34]. The cold exposure in the current study thus represented a metabolic challenge, which resulted in a highly significant 1.4 °C drop in the jackdaw’s cortical brain temperature. This drop in cortical temperature most likely reflected a cooler brain as a whole and perhaps a lower core body temperature as well. Studies in rats and rhesus monkeys have shown that cortical temperature is a good predictor for temperatures in deeper subcortical regions [35,36]. Also, in an earlier study in tupaias with a similar cold exposure paradigm, we found parallel drops in cortical brain temperature and core body temperature [22].

Importantly, and in contrast to many reports in mammals, sleep in our jackdaws was unaffected by cold exposure. Cold exposure during the nighttime sleep phase did not affect sleep quantity or sleep quality in terms of NREM EEG spectral power. It is particularly intriguing that cold exposure did not affect the amount of REM sleep in our jackdaws. Several studies have shown that a similar cold exposure results in a near-complete suppression of REM sleep in various species of mammals, including rats [18,19,20], cats [21], and tupaias [22]. This finding might suggest that the relationship between temperature regulation and REM sleep in birds and mammals is different. In fact, older studies in penguins showed a similar protection of REM sleep to cold exposure [25].

One difference between birds and mammals might be the degree to which they are able to regulate and control their metabolism and body temperature during REM sleep. A remarkable feature of REM sleep in mammals is the near-complete cessation of thermoregulation during this state [16,17]. Several studies have shown that the normal thermoregulatory responses that occur during wakefulness and NREM sleep, such as shivering and panting, are largely absent during REM sleep [16,23]. The current data in jackdaws could suggest that such a loss of thermoregulation may not occur during REM sleep in birds. Even though the average brain temperature of the birds was decreased during the cold night, the REM sleep episodes were associated with an increase in brain temperature rather than a decrease. While this does not prove the regulation of temperature during REM sleep, it does not support a loss of thermoregulation either.

One possible means of thermoregulation that may occur in birds but is largely absent in mammals is through muscle activity. While in mammals REM sleep is characterized by the complete loss of muscle tone, in birds the muscle atonia is often only partially expressed [11]. Birds are capable of engaging in REM sleep while maintaining body postures that require some degree of muscle activity, including the upright position of the head or even standing [11,13,37]. Yet, it remains to be established whether muscle activity truly contributes to heat production during REM sleep. For example, unlike mammals, birds can produce heat through the shivering of the pectoral muscle which is rich in specialized muscle fibers that can sustain activity for long periods of time [38]. The enhanced shivering of the pectoral muscle during cold exposure could be one mechanism through which birds produce heat, stabilize brain temperature, and maintain normal amounts of REM sleep without the risk of cooling down that occurs in mammals under these conditions. Our nuchal EMG recordings were unable to detect pectoral muscle activity, and therefore pectoral EMG recordings during cold exposure should be performed to support this hypothesis.

The modest increase in brain temperature over the course of REM sleep episodes we found in the jackdaws is in agreement with other studies in birds [33,39]. Instead, during NREM sleep episodes, the brain temperature in most cases remained stable but gradually decreased, particularly with long episodes. An earlier study in pigeons showed a similar decrease in brain temperature as in our jackdaws for the short NREM sleep episode [39], whereas a report in rooks showed a similar decrease in temperature as we reported in the jackdaw for the longer NREM sleep episodes up to 570 s [33].

Interestingly, although one might think that waking is a state of higher metabolic activity and higher brain temperature than sleep, the nighttime waking episodes in the jackdaws were most often associated with an initial steep drop in brain temperature, not only during the baseline night but even more so during the cold night. Here, the brain temperature decreased by about half a degree Celsius and only stabilized and increased again when episodes lasted longer than 75 s. The latter suggests that there is a delayed thermoregulatory response in waking episodes, which is not uncommon and depends on whether wakefulness is preceded by REM or NREM sleep [40].

## 5. Conclusions

In summary, while exposure to low ambient temperature posed a metabolic challenge and caused a significant drop in brain temperature in our jackdaws, it did not affect sleep time and sleep architecture. Most importantly, low ambient temperature was not associated with the suppression of REM sleep that has been reported for various mammalian species. These findings might suggest that thermoregulatory processes and/or REM sleep regulatory mechanisms in birds are different from those in mammals.

## Data Availability

Data are available upon request.

## Supplementary Materials

The following supporting information can be downloaded at https://www.mdpi.com/article/10.3390/biology13040229/s1, Figure S1: Example of brain temperature trace filtering.
