# Supplementary material for: Sleep and Thermoregulation in Birds: Cold Exposure Reduces Brain Temperature but Has Little Influence on Sleep Time and Sleep Architecture in Jackdaws (Coloeus monedula)
Source: Biology (Basel). 2024 Mar 29;13(4):229. doi: 10.3390/biology13040229 (PMC11047831; doi:10.3390/biology13040229)
Supplement: Supplementary file 1 [file biology-13-00229-s001.zip › biology-2874396-supplementary.pdf]

Sleep and thermoregulation in birds: cold exposure reduces brain temperature but has little influence on sleep time and sleep architecture in Jackdaws (*Coloeus monedula*)

Sjoerd J. van Hasselt[1], Massimiliano Coscia[1], Giancarlo Allocca[2,3], Alexei L. Vyssotski[4], Peter Meerlo[1*]

[1]Groningen Institute for Evolutionary Life Sciences, University of Groningen

[2]School of Biomedical Sciences, University of Melbourne, Parkville, VIC 3010, Australia

[3]Somnivore Pty. Ltd., Bachhus Marsh, VIC 3340, Australia

[4]Institute of Neuroinformatics, University of Zurich and Swiss Federal Institute of Technology (ETH), Zurich, Switzerland

[*] p.meerlo@rug.nl

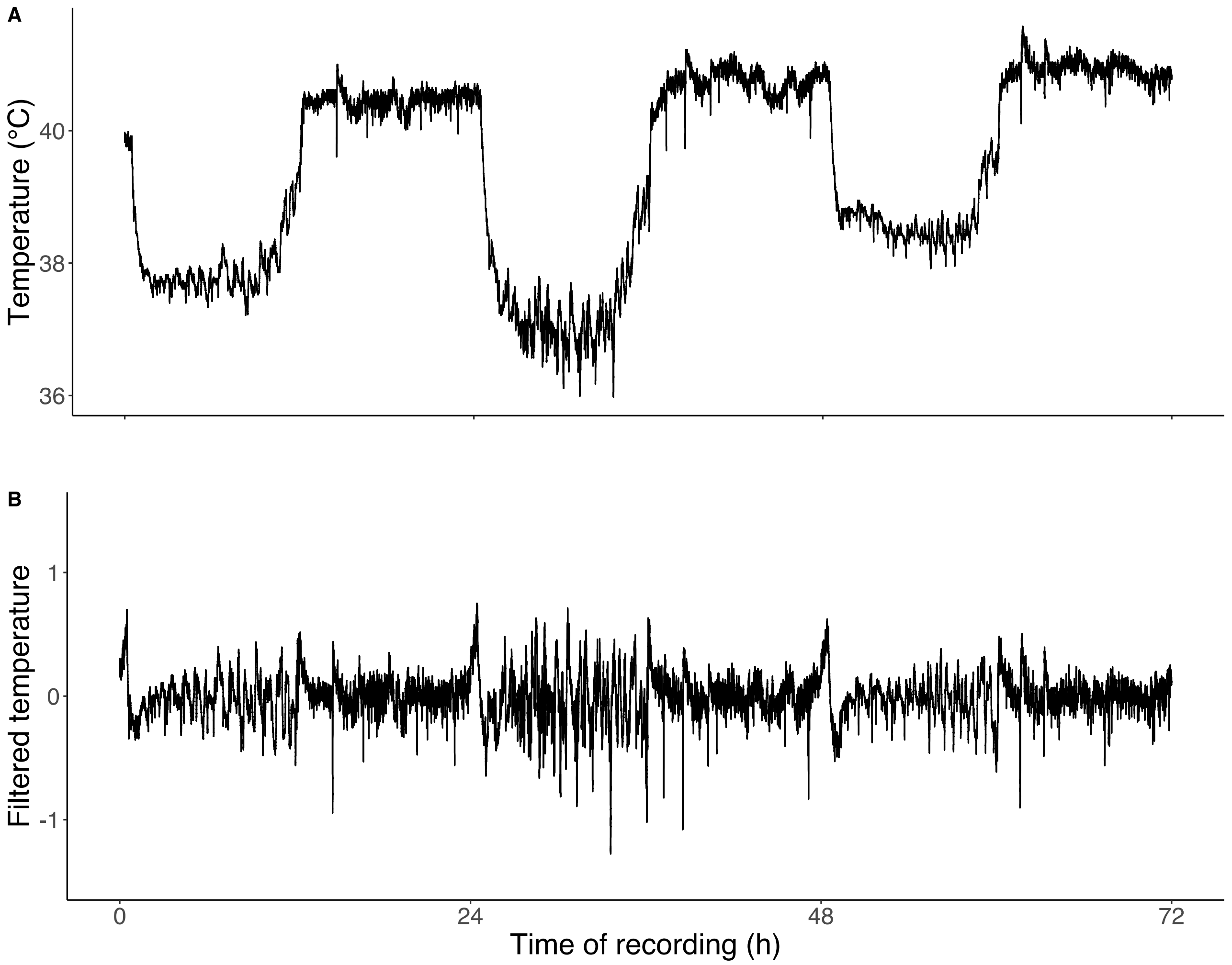

Figure S1. Example of brain temperature trace filtering. The raw temperature data (panel A) were subjected to a first-order bandpass Butterworth filter that removed waves with a period above 23.15 h (0.000012 Hz). The filtering successfully eliminated circadian temperature fluctuations in brain temperature while retaining faster stage-dependent fluctuations (panel B).
